# Improving medication management via dedicated smartphone app

**DOI:** 10.51866/lte.567

**Published:** 2024-01-21

**Authors:** Khaled Moustafa

**Affiliations:** 1 The Arabic Preprint Server (ArabiXiv), Paris, France. Email: khaled.moustafa@arabixiv.org

**Keywords:** Medication management, Digital health, Smartphone application for health, QR codes for health, Healthcare applications

**Dear editor,**

The widespread use of smartphones, often associated with a potential form of addiction,^1^ reflects the increasing integration of smartphone applications into both personal and professional lives.^2^ In various domains, ranging from medicine and social sciences to physics, chemistry, biology, geology and beyond, there is an upsurge of specialised smartphone applications designed to assist users in understanding and managing a spectrum of scientific concepts and information.

In light of the abovementioned context, I propose the development of a smartphone application and a dedicated Quick Response (QR) code (or other code specifically designed for the purpose described herein) that can be placed on medication boxes. Patients could then simply scan the code to access comprehensive and simplified information about their medications, including the uses, potential side eflects, dosage information, expiration date and other relevant information. Such a digital solution would be complementary to print leaflets and would make medication management easier and efficient with multiple benefits for patients.

First, the application would provide patients with easy access to important information about their medications. Currently, patients either rely on medication leaflets or search online for information, but they may have trouble understanding the complicated language found on medication labels. Moreover, medication packaging often lacks information regarding what a medication is used for or what disease it treats. Patients may also misplace their prescriptions or medication instructions, causing further confusion. Patients need to open and read medication inserts to learn about their medications, which can be time consuming and daunting. Having quick access to medication information through a simple click and a dedicated smartphone application would simplify this process. By scanning codes on medication boxes, patients could retrieve and easily comprehend important and accessible information about their medications, making managing their health simpler.

Second, the application could help reduce the risk of medication errors. In certain cases, patients may need to take multiple medications at the same time, which can lead to mixing up of one medication with another, especially if such medications look similar or if patients have memory or vision troubles. For instance, old people with multiple health conditions who take several medications may find it challenging to remember all the diflerent medications and their dosages, increasing the risk of accidentally taking the wrong medication or the wrong dosage, which can have serious consequences on their health. With an easily accessible application providing detailed vocal and/or textual information about each medication, patients could avoid or reduce such errors. By scanning QR codes on medication boxes, patients could access information about each medication’s uses, dosages and recommended schedules for intake. This would help ensure that they take the right medication at the right dosage and at the right time.

Finally, adding vocal guidance or instructions could further enhance the application’s versatility and usefulness. For example, the application could remind patients with impaired vision and hearing about a medication, its dosage and the number of times it should be taken through vocal prompts, triggered either after scanning the QR code or at the scheduled time prescribed by physicians.

However, such a solution is not without challenges. First, it would require tight cooperation between medication manufacturers and application developers to ensure that the information provided is accurate and up-to-date. Second, there would need to be a standard QR code format or other new adapted codes, such as visual scannable codes for direct transfer of information between two screens or documents to screens^3^, that all pharmaceutical companies would use so that the application could scan any medication box and provide the relevant information. Nevertheless, despite these minor issues, the benefits of a medication information application would likely outweigh the challenges from a long-term perspective. Unlike wearable health devices, which may raise some privacy concerns for patients, a smartphone application dedicated for medication management should not present such issues, as no personal information should be required to use it. Its primary goal must be to provide information about medications, not about patients.
